# Asia Pacific Delivery Science Conference (APDSC) 2024 co-organised by the CRS Malaysia, Australia, Chinese Taipei and New Zealand Local Chapters

**DOI:** 10.1007/s13346-025-01958-x

**Published:** 2025-08-21

**Authors:** Choon Fu Goh, Ju Yen Fu, Tushar Kumeria, Jui-Yang Lai, Yunching Chen

**Affiliations:** 1 Discipline of Pharmaceutical Technology, School of Pharmaceutical Sciences, Universiti Sains Malaysia, Penang, 11800 Malaysia; 2 Product Development and Advisory Department, Malaysian Palm Oil Board, 6 Persiaran Institusi, Bandar Baru Bangi, Kajang, 43000 Selangor Malaysia; 3 School of Materials Science and Engineering, University of New South Wales, Sydney, NSW 2052 Australia; 4 Department of Biomedical Engineering, Chang Gung University, Taoyuan, 33302 Taiwan; 5 Institute of Biomedical Engineering, National Tsing Hua University, Hsinchu, 30013 Taiwan

The Malaysia Local Chapter of the Controlled Release Society (CRS) has collaborated with the CRS Australian, Chinese Taipei and New Zealand Local Chapters in 2024 to organize Asia Pacific Delivery Science Conference (APDSC). APDSC 2024 celebrates a decade of excellence of the Malaysia Local Chapter of CRS (MyCRS), which was established in 2014 with the aim of providing a forum for researchers in drug delivery and controlled release to exchange ideas and learn from one another. In recognition of this milestone, APDSC 2024 stands as a testament to our commitment to push boundaries via fostering collaboration with the CRS Australian, Chinese Taipei and New Zealand Local Chapters. APDSC provides unique opportunities for the strong and rapidly growing delivery science community in the Asia Pacific region to build an understanding of how global innovations and technology advancements are shaping future solutions in delivery system. In line with the theme “Revolutionising Delivery System: Bridging Theory, Innovation, and Future Solutions”, the conference provides a platform for scientists and experts from universities, research institutions, and pharmaceutical and biotechnological industries in the field of delivery science and controlled release to share insights on the latest developments in biomaterials, food, pharmaceutical, cosmeceutical and nutraceutical technology.

In this Special Issue of Drug Delivery and Translational Research, we present a curated collection of 27 articles contributed by members of the participating CRS Local Chapters and APDSC 2024 attendees. This compilation encompasses a diverse range of original research and review articles, spanning macro to nano delivery systems, including hydrogels, RNA therapeutics, implants, microneedles, polymersomes, and lipid nanoparticles. More than just a showcase of pioneering research, this Special Issue underscores the power of interdisciplinary collaboration in advancing novel strategies to enhance drug delivery systems.

We envision this Special Issue as the starting point for fostering collaborative efforts among CRS Local Chapters across the Asia Pacific region and beyond. We firmly believe that the research presented here reinforces the groundbreaking ideas and scientific advancements that will propel us towards transformative solutions in healthcare.

